# The demic diffusion of Han culture into the Yunnan-Guizhou plateau inferred from ancient genomes

**DOI:** 10.1093/nsr/nwae387

**Published:** 2024-10-30

**Authors:** Kongyang Zhu, Changguo Hu, Meiqing Yang, Xinglong Zhang, Jianxin Guo, Mingxia Xie, Xiaomin Yang, Hao Ma, Rui Wang, Jing Zhao, Le Tao, Haifeng He, Wen Wan, Qun Zhang, Li Jin, Yunjie Zuo, Bisu Zhou, Jiang Huang, Chuan-Chao Wang

**Affiliations:** Key Laboratory of Environmental Pollution Monitoring and Disease Control, Ministry of Education, Department of Forensic Medicine, Guizhou Medical University, China; State Key Laboratory of Cellular Stress Biology, School of Life Sciences, Xiamen University, China; Guizhou Provincial Institute of Cultural Relics and Archaeology, China; Key Laboratory of Environmental Pollution Monitoring and Disease Control, Ministry of Education, Department of Forensic Medicine, Guizhou Medical University, China; Guizhou Provincial Institute of Cultural Relics and Archaeology, China; Department of Anthropology and Ethnology, Institute of Anthropology, Fujian Provincial Key Laboratory of Philosophy and Social Sciences in Bioanthropology, School of Sociology and Anthropology, Xiamen University, China; Key Laboratory of Environmental Pollution Monitoring and Disease Control, Ministry of Education, Department of Forensic Medicine, Guizhou Medical University, China; Department of Anthropology and Ethnology, Institute of Anthropology, Fujian Provincial Key Laboratory of Philosophy and Social Sciences in Bioanthropology, School of Sociology and Anthropology, Xiamen University, China; State Key Laboratory of Cellular Stress Biology, School of Life Sciences, Xiamen University, China; State Key Laboratory of Cellular Stress Biology, School of Life Sciences, Xiamen University, China; Department of Anthropology and Ethnology, Institute of Anthropology, Fujian Provincial Key Laboratory of Philosophy and Social Sciences in Bioanthropology, School of Sociology and Anthropology, Xiamen University, China; State Key Laboratory of Cellular Stress Biology, School of Life Sciences, Xiamen University, China; State Key Laboratory of Cellular Stress Biology, School of Life Sciences, Xiamen University, China; Key Laboratory of Environmental Pollution Monitoring and Disease Control, Ministry of Education, Department of Forensic Medicine, Guizhou Medical University, China; Department of Archaeology, School of History, Wuhan University, China; State Key Laboratory of Genetic Engineering, Collaborative Innovation Center for Genetics and Development, School of Life Sciences and Human Phenome Institute, Fudan University, China; Ministry of Education Key Laboratory of Contemporary Anthropology, Department of Anthropology and Human Genetics, School of Life Sciences, Fudan University, China; Guizhou Provincial Institute of Cultural Relics and Archaeology, China; Guizhou Provincial Institute of Cultural Relics and Archaeology, China; Key Laboratory of Environmental Pollution Monitoring and Disease Control, Ministry of Education, Department of Forensic Medicine, Guizhou Medical University, China; State Key Laboratory of Cellular Stress Biology, School of Life Sciences, Xiamen University, China; Department of Anthropology and Ethnology, Institute of Anthropology, Fujian Provincial Key Laboratory of Philosophy and Social Sciences in Bioanthropology, School of Sociology and Anthropology, Xiamen University, China; Ministry of Education Key Laboratory of Contemporary Anthropology, Department of Anthropology and Human Genetics, School of Life Sciences, Fudan University, China

According to the historical records of *Records of the Grand Historian* (史记) and genomic evidence, the Han Chinese could trace their ancestry back to the Huaxia tribe, people who lived along the Yellow River basin since Neolithic times [1]. From this region, Han culture spread west into the Tibetan Plateau and south into southwest China over the past two millennia. For the Han population, on the one hand, evidence from classical genetic markers and microsatellites observed the differentiated groups between northern Han and southern Han, seeming to support the culture diffusion model of Han expansion [2,3]. On the other hand, archaeological and genomic studies show that the millet agricultural population originated in the Yellow River basin area and expanded outward ∼5400 years ago [4], which not only spread millet agriculture but also contributed to the formation of present-day Sino-Tibetan–speaking populations in this region [5,6]. The study based on Y-chromosomes and mitochondrial DNA (mtDNA) from modern Han populations also suggests that the massive movement of the northern Han resulted in the demographic expansion of Han populations and their culture [7]. Moreover, the previous study of ancient genomics from Gaoshan and Haimenkou sites also supports the co-diffusion model of millet farming culture and population from the Yellow River basin into the Sichuan and Yunnan areas of southwest China [8]. In the historical period, several large-scale migrations of Han people from the north to the south, according to records, have further shaped the genetic landscape of present-day southern Han Chinese [9]. The previous study has found that ancient groups from Guangxi about 500∼1500 years ago had closer genetic links with Tai-Kadai and Hmong-Mien populations, providing genetic evidence for the formation of these populations in southwestern China [10]. However, so far, ancient human genomes from sites containing the influence of Han culture from the historical period are still lacking, hindering the comprehensive understanding of the expansion process of Han culture into this region.

Here, we report genome-wide data of 99 ancient individuals (Table S1A) from the Songshan site in Guizhou province, southwest China (see Supplementary Materials for details). The time span of calibrated carbon dating ranges from 990 AD to 1649 AD (Table S1B), covering the period from the Song Dynasty to the Ming Dynasty. Our result shows that the Songshan populations are different from historical ancient individuals from Guangxi [10], showing a genetic makeup of major Yellow River-related ancestry shared in the Han population. This suggests that the expansion of Han culture was accompanied by the migration of the Han population. Our result supports the demic-diffusion model of the expansion of Han culture to southwest China from the perspective of ancient genomics.

After quality control (see Supplementary Materials for details), 57 ancient individuals were used for downstream analyses with 15 964–582 350 and 31 116–1 192 118 SNPs genotyped on HO and 1240k panels, respectively (Table S1A, Table S2, Figs. S1–S4). Since the calibrated carbon dating time spans a period of ∼700 years, covering the period from the Song Dynasty to the Ming Dynasty, we first checked the consistency of genetic profiles of Songshan individuals across different periods. The results from qualitative principal component analysis (PCA), quantitative *f*_4_-statistics in the format of *f*_4_(Mbuti, References; Songshan1, Songshan2) and pairwise-*qpWave* test suggest that Songshan individuals shared similar genetic profiles crossing the long time span except for four outliers from the Ming Dynasty (Figs. S5–S7, Table S3). Hence, we analyzed these individuals as one population in the following analyses. The ancient individuals from the Songshan site could be divided into three sub-groups: Songshan, Songshan_o1 and Songshan_o2 (Fig. 1A). Songshan, which represents the main genetic compositions of the ancient Songshan individuals, was projected between ancient Yellow River (YR) populations and ancient Southeast Asia (SEA) populations (Fig. 1A), and fall in the Han population cline with southern Han (Han_Fujian and Han_Guangdong) on the left and northern Han (Han_Shandong and Han_Shanxi) on the right, and clustered with center Han (Han_Hubei and Han_Chongqing) who are geographically closer to Guizhou (Fig. S8). Songshan_o2, composed of three ancient individuals, shows a closer genetic affinity towards modern Hmong-Mien speaking populations and clustered with GaoHuaHua, who are assumed to contribute to the present-day Hmong-Mien speakers (Fig. 1A, Fig. S8) [10]. Songshan_o1 is composed of only one ancient individual projected between Songshan and Songshan_o2. Similar patterns could be observed from the ADMIXTURE result and outgroup-*f*_3_ analyses (Figs. S9–S11), where Songshan shared more genetic drift with Han_Hubei and Han_Chongqing and Songshan_o2 shared more genetic drift with modern Hmong-Mien speaking populations and ancient Guangxi GaoHuaHua (Fig. S11).

**Figure 1.**
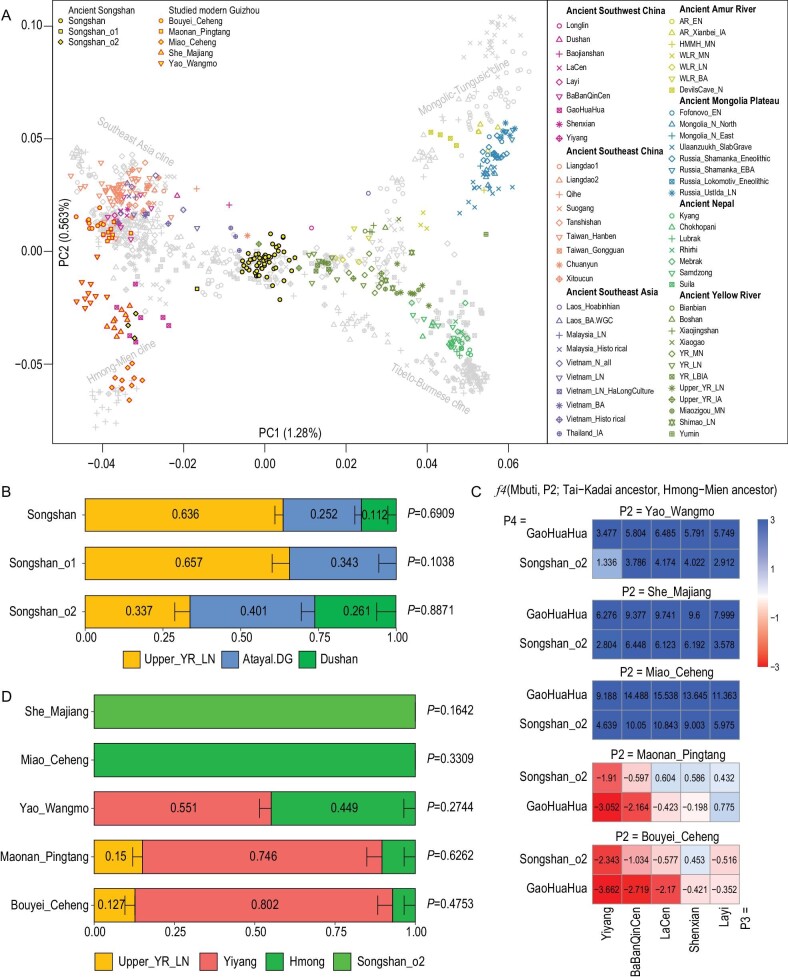
(A) PCA result of Songshan individuals and studied modern Guizhou populations with present-day and ancient East Asians. (B) Ancestry proportion estimated for Songshan populations via qpAdm (see Table S5 for details). Error bars represent one standard deviation. (C) *F*4-statistics test in the format of *f*4(Mbuti, X; Tai-Kadai ancestor, Hmong-Mien ancestor) indicating the close genetic affinity between Tai-Kadai speaking Bouyei Maonan with potential Tai-Kadai ancestor (Songshan_o2, GaoHuaHua), and the close genetic affinity between Hmong-Mien speaking Miao, She and Yao with potential Hmong-Mien ancestor (Yiyang, BaBanQinCen, LaCen Shenxian and Yiyang), |Z|≥3 was coloured by deep red or deep blue, 3>|Z|≥2 was coloured by light red or light blue. *Z*-scores are labeled in the center of squares. (D) Ancestry proportion estimated for studied modern Guizhou populations qpAdm (see Table S5 for details). Error bars represent one standard deviation.

Next, we investigated the fine-scale genetic profiles of the Songshan populations using quantitative *f*-statistics–based *f*_4_ tests and *qpAdm* analyses. We first investigated the genetic influence of SEA-related deep ancestry in Songshan populations. Compared to other ancient individuals from East Asia, the Songshan populations shared similar genetic affinity with the Hòabìnhian-related hunter-gatherer lineage shown by non-significant *Z*-scores of *f*_4_(Mbuti, Hòabìnhian; Songshan populations, ancient East Asia) (Table S4A, B). However, they showed a significant genetic affinity with ancient Guangxi ancestry (Longlin, Dushan and Baojianshan) [10], especially with Dushan shown by *f*_4_(Mbuti, Guangxi ancestry; Songshan populations, ancient East Asia) (Table S4C–E). We further performed *f*_4_(Mbuti, Songshan populations; ancient Guangxi1, ancient Guangxi2) to investigate the ancient Guangxi individuals that shared the closest genetic affinity with the Songshan populations (Table S5A). We found that Songshan and Songshan_o2 shared the closest genetic affinity with Dushan, shown by *f*_4_(Mbuti, Songshan; Dushan, Longlin/Baojianshan), i.e. *Z*-scores = −7.17 and −3.79 and *f*_4_(Mbuti, Songshan_o2; Dushan, Longlin/Baojianshan), i.e. *Z*-scores = −3.35 and −3.52, respectively. While they showed a non-significant genetic affinity with Longlin and Baojianshan populations, shown by *f*_4_(Mbuti, Songshan/Songshan_o2; Baojianshan, Longlin), i.e. *Z*-scores = −2.88 and −0.42. Thus, we used the Late Neolithic Upper Yellow River population (Upper_YR_LN) as a YR-related source, Atayal as a SEA-related source and Dushan as an ancient Guangxi source to model the Songshan populations (Fig. 1B, Table S5B). The result showed that Songshan could be modelled as deriving 63.6% from YR-related ancestry, 25.2% from SEA-related ancestry and 11.2% from ancient Guangxi ancestry. Songshan_o2 could be modelled as deriving more ancestry from ancient Guangxi, as 33.7% from YR-related ancestry, 40.1% from SEA-related ancestry and 26.1% from ancient Guangxi-related ancestry. Songshan_o1 was modelled as not deriving ancestry from ancient Guangxi-related ancestry, as 65.7% from YR-related ancestry and 34.3% from SEA-related ancestry. Our results suggest that Songshan populations from the Song Dynasty to the Ming Dynasty are mainly composed of immigrated Han populations and mixed with local ancestry to varying degrees.

Here, we also report three Hmong-Mien speaking populations (She_Majiang, Miao_Ceheng and Yao_Wangmo) and two Tai-Kadai speaking populations (Bouyei_Ceheng and Maonan_Pingtang; see Supplementary Materials for details) from Guizhou province to investigate the contribution of Songshan populations to modern Guizhou ethnic groups. In PCA results, two Tai-Kadai speaking populations, Bouyei_Ceheng and Maonan_Pingtang, tightly cluster with other modern Tai-Kadai speaking populations, with assumed Tai-Kadai ancestors projected close to them (Fig. S8). Two Hmong-Mien–speaking populations, She_Majiang and Miao_Ceheng, are projected on the modern Hmong-Mien cline. Interestingly, the Hmong-Mien–speaking Yao_Wangmo did not project on the contemporary Hmong-Mien cline but projected between the Tai-Kadai cluster and Hmong, suggesting the close genetic affinity with Tai-Kadai populations compared to Bouyei_Ceheng and Maonan_Pingtang. The result of *f*_4_(Mbuti, Songshan_o2/GaoHuaHua; GaoHuaHua/Songshan_o2, ancient Guangxi) suggests the close genetic affinity of Songshan_o2 with GaoHuaHua consistent with the result seen in PCA analysis (Table S5C). Thus, we used Songshan_o2 and GaoHuaHua as potential Hmong-Mien ancestors and other historical Guangxi individuals (Layi, Shenxian, LaCen, BaBanQinCen and Yiyang) as Tai-Kadai ancestors to investigate the genetic profiles of our studied modern Guizhou populations. The result of *f*_4_(Mbuti, studied modern Guizhou populations; Tai-Kadai ancestor, Hmong-Mien ancestor) suggests the genetic affinity of Hmong-Mien ancestor (GaoHuaHua and Songshan_o2) with Hmong-Mien speaking She_Majiang, Miao_Ceheng and Yao_Wangmo, and the genetic affinity of Tai-Kadai ancestor (Yiyang and BaBanQinCen) with Tai-Kadai speaking Maonan_Pingtang and Bouyei_Ceheng (Fig. 1C, Table S5D). We further modelled the studied modern Guizhou populations by *qpAdm*. Hmong-Mien–speaking She_Majiang and Miao_Ceheng could be modelled as deriving 100% from Hmong-related ancestry (Hmong or Songshan_o2), while Yao_Wangmo required extra Tai-Kadai–related ancestry consistent with PCA results. Tai-Kadai speaking Bouyei_Ceheng and Maonan_Pingtang could be modelled as deriving most of the ancestry (74.6%∼80.2%) from potential Tai-Kadai ancestors (represented by Yiyang) and the rest from a YR-related source and Hmong-related source (Fig. 1D).

Here we provide the first genome-wide data of ancient individuals from Guizhou Province dating back to 990 AD to 1649 AD across the Song Dynasty to the Ming Dynasty. Our results showed the genetic diversity and consistency of Songshan individuals across time, suggesting a long-term stable society in this region. We revealed their Yellow River–related genetic profiles, consistent with the historical records of massive Han immigration from north to south, supporting the demic-diffusion model of the expansion of Han culture to southwest China since the Han Dynasty. Moreover, we observed the genetic contribution of Hmong-Mien–related Songshan ancestry to modern Tai-Kadai– and Hmong-Mien–speaking populations, providing a genetic link of the Songshan site to present-day minorities in southwest China. Our study offers an insight into the genomic makeup of the historical Guizhou populations.

## ETHICS STATEMENT

Approval for the use of ancient human individuals was curated by co-authors and obtained with permission from the respective provincial archaeology institutes or universities that managed the samples.

## Supplementary Material

nwae387_Supplementary_Files

## Data Availability

The DNA sequences reported in this paper have been deposited in the Genome Sequence Archive (GSA) in the National Genomics Data Center under accession HRA006067, following the regulations of the Human Genetic Resources Administration of China (HGRAC).

## References

[1] Ning C, Li T, Wang K et al. Nat Commun 2020; 11: 2700. 10.1038/s41467-020-16557-2 32483115 PMC7264253

[2] Chu JY, Huang W, Kuang SQ et al. Proc Natl Acad Sci USA 1998; 95: 11763–8. 10.1073/pnas.95.20.11763 9751739 PMC21714

[3] Zhao T, Lee TD. Hum Genet 1989; 83: 101–10. 10.1007/BF00286699 2777248

[4] Social Sciences in China . Archaeological Presentation of the Origin of Civilization in the Central Plains. https://www.cssn.cn/skgz/bwyc/202208/t20220803_5459425.shtml (24 November 2023, date last accessed).

[5] Chen FH, Dong GH, Zhang DJ et al. Science 2015; 347: 248–50. 10.1126/science.1259172 25593179

[6] Wen B, Xie X, Gao S et al. Am Hum Genet 2004; 74: 856–65. 10.1086/386292 PMC1181980 15042512

[7] Wen B, Li H, Lu D et al. Nature 2004; 431: 302–5. 10.1038/nature02878 15372031

[8] Tao L, Yuan H, Zhu K et al. Curr Biol 2023; 3: 4995–5002.e7. 10.1016/j.cub.2023.09.055 37852263

[9] Ge JX, Wu SD. History of Migrations in China. Shanghai: Fudan University Press, 2022.

[10] Wang T, Wang W, Xie G et al. Cell 2021; 184: 3829–41. 10.1016/j.cell.2021.05.018 34171307

