# Supplementary material for: The demic diffusion of Han culture into the Yunnan-Guizhou plateau inferred from ancient genomes
Source: Natl Sci Rev. 2024 Oct 30;11(12):nwae387. doi: 10.1093/nsr/nwae387 (PMC11647586; doi:10.1093/nsr/nwae387)
Supplement: nwae387_Supplementary_Files [file nwae387_supplementary_files.zip › Supplementary methods.docx]

# MATERIALS AND METHODS

## Sample collection

This study collected dental elements from 99 ancient individuals housed at the Songshan site. Approval for their use was curated by co-authors and obtained with permission from the respective provincial archaeology institutes or universities that managed the samples. The permission and oversight were also provided by the institutional review board of the Ethics Committee for Biological Research at Xiamen University.

In this study, we also generated the genome-wide data of fifty-one unrelated modern individuals belonging to five ethnic groups (labeled as She_Majiang, Miao_Ceheng, Yao_Wangmo, Maonan_Pingtang and Bouyei_Ceheng) from four counties (Ceheng, Majiang, Wangmo and Pingtang) in Guizhou Province, including three Hmong-Mien speaking populations (Miao, She and Yao) and two Tai-Kadai speaking populations (Bouyei and Maonan) with written informed consent and performed whole genomic sequencing on the DNBSEQ-T7 platform. The study involving human participants was reviewed and approved by the Medical Ethics Committee of Guizhou Medical University and Xiamen University.

## Radiocarbon dating of sample materials

Given the practical constraints of dating all materials, we chose samples that, based on the archaeological context, were likely to span different periods of the site's occupation. We also selected teeth that were in good condition and likely to yield reliable radiocarbon dates. In total, eight human bone samples were analysed by AMS at Beta Analytic. Uncalibrated direct carbon dates were successfully obtained (Table S1B). The resulting ^14^C dates were calibrated using OxCal v4.4.4 [1] and the IntCal20 calibration curve [2].

## Ancient DNA extraction and library preparation

We screened a total of 99 samples from the Songshan site located in the Gui'an New Area, Guizhou Province. All samples were processed in the dedicated ancient DNA facility at the Institute of Anthropology, Xiamen University, following established precautions for working with ancient human DNA [3, 4]. The human remains were cleaned with 75% ethanol, and the surface was cleaned using a drill bit. Next, samples were washed with 10% NaClO and exposed to ultraviolet for 30 minutes. We collected 60-150 mg of bone powder by drilling deep into the dental pulp and the dense parts of the limb bone. After adding 1 mL of 0.5 mM EDTA and 0.25 mg/mL Proteinase K to the bone powder, the mixture was agitated at 300 rpm and incubated overnight at 37°C for lysis. After centrifugation, the precipitation was discarded, and 12.5 mL binding buffer was added to the supernatant. The binding buffer contains 5 M Guanidine hydrochloride, 40% Isopropanol, 25 mM sodium acetate, and 0.05% Tween-20 (Sigma Aldrich, Germany) at pH 5.5. Then, we purified samples using the MinElute kit (Qiagen, Germany) and eluted DNA extract by 0.1×TE. We used the NEBNext Ultra™ II DNA Library Prep Kit with an adaptor from blunt-ended ligation-based approaches [5] instead of a circular NEBNext Adaptor to prepare double-stranded libraries. Then, we purified the DNA library with the AMPure XP beads (Beckman Coulter, USA) and employed the conventional agarose gel electrophoresis method to inspect the library strips. We employed hybridisation capture using the Twist Ancient DNA protocol developed by David Reich's lab [6], finished by purifying the PCRs with 1.8× AMPure XP beads (Beckman Coulter, USA). Finally, sequencing was performed on the Illumina NovaSeq platform.

## DNA extraction, library preparation and sequencing of present-day populations

Genomic DNA from present-day populations in Guizhou was extracted using the PureLink Genomic DNA kit (Thermo Fisher Scientific). Following the kit's instructions, the extraction was performed automatically by KingFisher Flex and the DNA was ultimately dissolved in an appropriate eluent. DNA concentrations were measured using a Qubit 4.0 fluorometer. Electrophoresis was conducted on 1% agarose gel to ensure that most genome fragments had major peaks larger than 20Kb and were not significantly degraded.

Whole genome DNA was randomly fragmented using an ultrasonic fragmentation instrument. DNA fragments with a main peak of 300-350bp were selected using magnetic beads. The DNA fragments underwent end-repair, and a dATP was added to the 3'-end. The ends of the repaired DNA fragments were then ligated to adapters suitable for sequencing anchoring. Finally, PCR amplification was performed. The qualified PCR products were separated by thermal denaturation at 95°C. Circularization was carried out to prepare single-stranded circular DNA (ssCir DNA), generating the final library.

DNA nanospheres (DNBs) were prepared by rolling circle amplification of qualified libraries. The DNBs were loaded and anchored to the chip, and sequencing was conducted on the DNBSEQ platform using the PE150 bp sequencing strategy. The final data files were stored in FASTQ format.

## Sequence data processing

For ancient genome-wide data, AdapterRemoval v2.3.1 [7] was performed to trim the sequencing adapters and merge the paired-end reads into a single sequence. Reads overlapped by at least eleven bp were retained and mapped onto the human reference genome (hs37d5; GRCh37 with decoy sequences) using BWA v0.7.17 [8] samse, with parameters -n 0.01 and -l 1024. Dedup v0.12.3 [9] was used to remove the PCR duplicates. To avoid excess C->T and G->A transitions at the ends of sequences, ten bases were clipped from both ends using trimBam implemented in BamUtil v1.0.14 (<https://github.com/statgen/bamUtil>). We filtered alignment quality using mpileup implemented in samtools with parameters -q30 and -Q30 before generating pseudo-haploid calls for our ancient individuals using parameter --RandomHaploid in pileupCaller software (<https://github.com/stschiff/sequenceTools>) and the 1240k dataset as reference.

For modern genome-wide data, reads were mapped to the human reference genome (hs37d5; GRCh37 with decoy sequences) using BWA mem. We used Dedup v0.12.3 [9] to remove the PCR duplicates and then generated genome-wide SNP data on a 1240k panel using gatk HaplotypeCaller [10].

## Authentication of Ancient DNA

Deamination patterns of ancient DNA (5’ C>T and 3’ G>A misincorporation) were analysed using pmdtools [11] (<https://github.com/pontussk/PMDtools>). We then employed schmutzi v1.5.5.5 to estimate mtDNA contamination rates [12] and used ANGSD v0.910 [13] to estimate the nuclear genome contamination rate for all male individuals.

## Genetic sexing and uniparental haplogroup assignment

We compared the genome coverage of the X and Y chromosomes to autosomes [34] to determine the genetic sex of ancient individuals. We utilised the log2fasta program implemented in Schmutzi [12] to call the mtDNA consensus sequences, and the mitochondrial haplogroups were assigned using Haplogrep2 [14]. Yleaf [15] was used to assign Y chromosome haplogroups.

## Kinship detection

We used lcMLkin [16] to detect the genetic relationship between ancient individuals. Six pairs of individuals under second degree were detected and labeled with the suffix “_kin.”

We used King [17] to detect the relationship between modern individuals with parameters --related, --ibs and --degree 2. No pair of individuals under the second degree was detected.

## Data merging

We merged our data with two previously published datasets [18] using mergeit, which was implemented in EIGENSOFT [19]. The “Human Origin” dataset contained 597,573 SNPs and was used in smartpca, ADMIXTURE and outgroup-*f*_3_ analyses. The Illumina array “1240k” dataset containing 1,233,013 SNPs was used for *f*-statistics-based analyses.

## Principal Components Analysis

We performed principal components analysis (PCA) analysis on the Human Origin dataset using smartpca v16000 implemented in EIGENSOFT. The default parameters were used, and lsqproject was set to YES [19]. The modern populations were used to calculate the principal components (PCs), and then we projected the ancient individuals onto the top two components. A total of 473,720 SNPs were used to calculate the PCs.

## ADMIXTURE Analysis

After pruning for linkage disequilibrium in plink v1.90 [20] using parameters --indep-pairwise 200 25 0.4 [21, 22], 209,588 SNPs were used to perform an unsupervised admixture analysis using ADMIXTURE v1.3.0 [23] with parameters five-fold cross-validation, 100 bootstrap replicates, and varies of ancestral populations K ranging from 2 to 6.

## *F*-statistics

We used *qp3Pop* v651 to calculate the outgroup-*f*_3_ using parameter inbreed: YES and performed *qpDstat* v980 to calculate the *f_4_*-statistics using parameter *f_4_*-mode: YES [21, 24]. Both software are implemented in ADMIXTOOLS [24].

## Admixture modelling

*QpAdm* v810 implemented in ADMIXTOOLS was used to estimate the ancestry proportion of studied populations as the combination of the source populations [24]. Specifically, we set the parameters allsnps: YES and inbreed: YES, and used the rotation strategy described in Harney et al. [25]. A set of fixed outgroups consisting of distantly related individuals was used to model our samples as the combination of one to three potential sources, and a population was added to the outgroup whenever it was not chosen as a potential source.

# Supplementary Text

## Archaeological information

Guizhou, located in southwest China, is situated on the Yunnan-Guizhou Plateau and has a complex environment. The relatively isolated environments have resulted in diverse populations and languages in Guizhou. Populations speaking Tai-Kadai, Hmong-Mien and Sino-Tibetan languages have settled here, which makes Guizhou present multilingual, multicultural and multiethnic patterns. The Songshan site is in the eastern Yunnan-Guizhou plateau in Machang Town, Gui'an New Area, in the central region of Guizhou Province. From July 2022 to January 2023, the Guizhou Institute of Cultural Relics and Archaeology, together with Peking University, Sichuan University and Sun Yat-sen University, carried out a comprehensive archaeological excavation of this burial site. This excavation covers an area of 13,500 square meters, and a total of 2,192 tombs were cleared. Besides, more than 4,000 pieces of various cultural relics were unearthed.

The Songshan cemetery spans from the Two Jin Dynasties to the Ming Dynasties. It offers valuable insights into various aspects of life, trade, faith, funerary practices, and more throughout different historical periods in this region. This archaeological site serves as a testament to the rich history, culture, and civilization of the ancient inhabitants of the southwest frontier for over 1,400 years. The tombs also reflect the development process of ancient culture and civilization in central Guizhou. The unearthed artefacts reflect the apparent Han cultural influence on this area throughout the development and evolution of this cemetery (see Archeological information section in Supplementary materials online), which is consistent with historical records of several southward migrations since the Han Dynasty [26], providing a perfect chance to directly test the demic or cultural diffusion models of Han culture.

The Songshan cemetery can be divided into two phases. The early phase was from the Two Jin Dynasties to the Tang Dynasties. A total of 155 tombs during this period were excavated. These tombs are primarily located in the sloping terrain surrounding the Songshan reservoir, suggesting a pattern resembling small family burial grounds. The late phase corresponds to the Song, Yuan, and Ming Dynasties and includes a total of 2,037 tombs. These tombs can be categorized into two main types: stone chamber tombs and earthen pit tombs. The distribution of tombs shifted gradually from the higher eastern side to the lower western and northwestern areas over time, forming a layout characteristic of a communal cemetery.

The excavated artifacts from the Songshan cemetery reveal a pronounced cultural transition between the early and late phases. In the early phase, there was a prevailing influence of Han culture, complemented by a secondary presence of regional ethnic culture. Among the burial goods, there appears a significant number of Han culture-related items such as porcelain and bronze mirrors. Conversely, the late phase is marked by the predominance of regional ethnic culture, accompanied by a secondary influence of Han culture. Traditional elements such as beads, bells, copper hairpins and silver comb ornaments, tubular copper neck ornaments, intricately patterned copper rings and bracelets, as well as glazed pottery jars with ox horn patterns, all reflect strong characteristics of regional ethnic culture. These artifacts also reflect a religious belief system that places equal importance on Confucianism, Buddhism, and Taoism. Throughout the development and evolution of the Songshan cemetery, elements of Han culture have consistently held a significant role.

## Ancient genome data production and quality control

This study generated the genome-wide data of 99 ancient individuals from the Songshan site using an in-solution hybridization capture approach (Twist ancient panel) to enrich the endogenous DNA. We then generated genome-wide data of these individuals from the Illumina NovaSeq platform. The authenticity of genome-wide data was checked by verifying the presence of characteristic post-mortem patterns of ancient DNA (Table S1 and Table S2, Fig. S1-4). Four individuals with mtDNA contamination larger than 2%, estimated by Schmutzi, and one with nuclear genome contamination larger than 2%, estimated by ANGSD, were labelled with the suffix “_cont” and excluded from subsequent analyses (Table S1A). The kinship between individuals was estimated using lcMLkin, in which six pairs of kinship under second-degree were detected, and individuals with lower coverage in each pair were labelled with the suffix “_kin” and excluded from subsequent analyses (Table S1A, see Materials and Method section in Supplementary materials online). We then trimmed ten bp from each end of the reads that mapped to the human reference genome (hs37d5; GRCh37 with decoy sequences) and generated pseudo-haploid data using two single nucleotide polymorphisms (SNPs) panels: Affymetrix “Human Origin” (HO) [24], and Illumina “1240k” [27-29]. The SNP coverage on 1240k lower than 30,000 was labelled with the suffix “_lc” and excluded from downstream analyses (Table S1A). As a result, 57 ancient individuals were used for downstream analyses with 15,964-582,350 and 31,116-1,192,118 SNPs genotyped on HO and 1240k panels, respectively (Table S1A). We then merged these data with previously published ancient and modern genomic data for further analyses [30]. In this study, we also report genome-wide data of three modern Hmong-Mien speaking populations and two modern Tai-Kadai speaking populations from Guizhou.

## Authentication of ancient DNA

The read length of our DNA libraries shows a bias of enriching long reads, which was not observed in our other research. To investigate whether this unusual pattern was caused by samples or our experimental procedure, we used the same bottle of AMPure XP (Beckman Coulter, USA, lot 19820000) used in this study to purify 100bp DNA ladder (Takara Bio, Japan). We found decreased DNA recovery rates on 100bp DNA based on agarose gel electrophoresis (AGE) analysis (Fig. S1), and that was consistent with our observation of DNA selection causing the absence of 100bp~200bp DNA that contains 106bp adapter and sample DNA range from 0 to 100bp (Fig. S3). Since our 2 batch experiments with different samples show similar patterns of DNA size selection, we assumed that our newly arrived AMPure XP (Beckman Coulter, USA) used in those studies lost the ability to recover DNA shorter than 200bp. We ordered another AMPure XP (Beckman Coulter, USA, lot 19936700) and used it to purify a 50bp DNA marker (Tiangen, China) to testify to the ability of DNA recovery. Our result shows that lot 19936700 can bind 150bp and 200bp DNA at similar levels, which was sufficient for PCR cleanup (Fig. S2). We further tested this reagent using other samples with the remaining reagents unchanged, and results from mapDamage show no size selection was observed like before (Fig. S3). Thus, we believe that the abnormal size selection observed in this study was caused by the reagent AMPure XP (Beckman Coulter, USA, lot 19820000).

To test the consistency of genetic information from different read lengths from the same samples, we performed *f*-statistics in the form of *f_4_*(Mbuti, References; X, Y) on four high-coverage individuals. Where we filtered the sample reads by different read lengths (30-80bp, 80-130bp, 130-180bp) and damage patterns (dam). Our results show that non-significant Z-scores were observed in most of the *f_4_* tests (Table S2).

## Haplogroup analysis of modern Guizhou populations

Analysis of mtDNA and Y-chromosome haplogroups reveals distinct patterns between the ancient Songshan and modern Guizhou populations. The Songshan population exhibited dominant mtDNA haplogroups B4c2c (48.4%) and M7b1a1a3 (15.4%), while their Y-chromosome haplogroups were predominantly various subclades of haplogroup C2b1b1-Z12272 (77.8%) (Table S1A). In contrast, modern Guizhou ethnic groups display a more diverse distribution of mtDNA haplogroups (Table S1E). Interestingly, 35.1% of the haplogroup types found in modern populations were also present in the Songshan population, suggesting a degree of genetic contribution from the ancient Songshan people to contemporary ethnic groups in Guizhou. The Y-chromosome haplogroup distribution in modern populations differs from that of the Songshan population and varies considerably among different ethnic groups, with high inner-group homogeneity (Table S1E). All detected Bouyei_Ceheng Y-chromosome lineage belong to the haplogroup O1b1a1a1-M1348, while all Miao_Ceheng samples fall under O2a2a1a2a1a1a2a1a1a1-F22246. The Maonan_Pingtang samples predominantly belong to various subclades of the haplogroup O1b1a1a1a1a1a-Z24085 (83.3%). Notably, the She_Majiang population exhibits a distinct pattern with a high frequency of the haplogroup N1a1a1a1a-M2034 (87.5%), differentiating them from both the ancient Songshan population and other modern groups in this study. These haplogroup patterns provide nuanced insights into the genetic history of the region. The shared mtDNA haplogroups between the ancient Songshan population and modern groups support some level of genetic continuity. However, the stark differences in Y-chromosome haplogroups indicate significant male-mediated gene flow and population replacements over time.

## Limitations of this study

Although we used the Yellow River millet farmers as the genetic surrogate of ancient Han Chinese in modelling the formation of Songshan samples, we cannot directly infer the diffusion of millet agriculture to Guizhou was derived by population migration using our current genomic data from the Song to Qing dynasties (990 to 1649 AD), because the spread of millet agriculture in southwestern China can be traced back to the earlier late Neolithic period. We note that our study has limitations due to the lack of high-coverage genomic data from both ancient Songshan individuals and present-day Han Chinese populations in southwest China. At our current resolution, we cannot perform more fine-grained analyses to distinguish between the genetic influences of the main Songshan population and Yellow River basin populations on modern populations in southwest China. This limitation prevents us from fully exploring the potential genetic contribution of Songshan individuals to present-day Han groups in Guizhou and other parts of southwest China, which would be crucial for a comprehensive understanding of Han culture expansion. Further studies with more comprehensive datasets will be necessary to address these questions. Additionally, investigations on the early phase of the Songshan cemetery will provide additional insights into the formation and development of the Guizhou population.

# Supplementary Figures


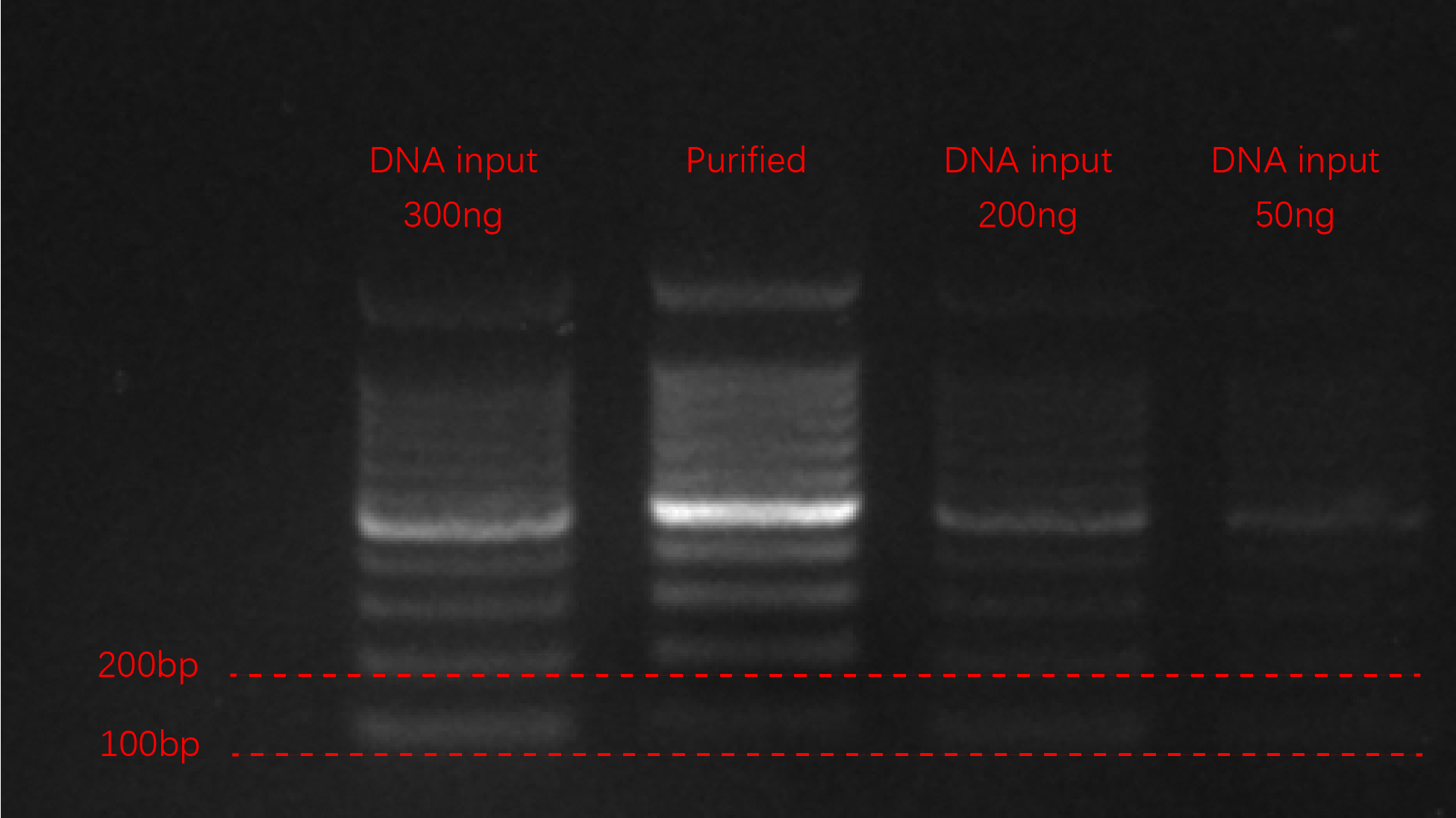


**Fig. S1** DNA library prepared using AMPure XP (Beckman Coulter, USA, lot 19820000, used in this study).


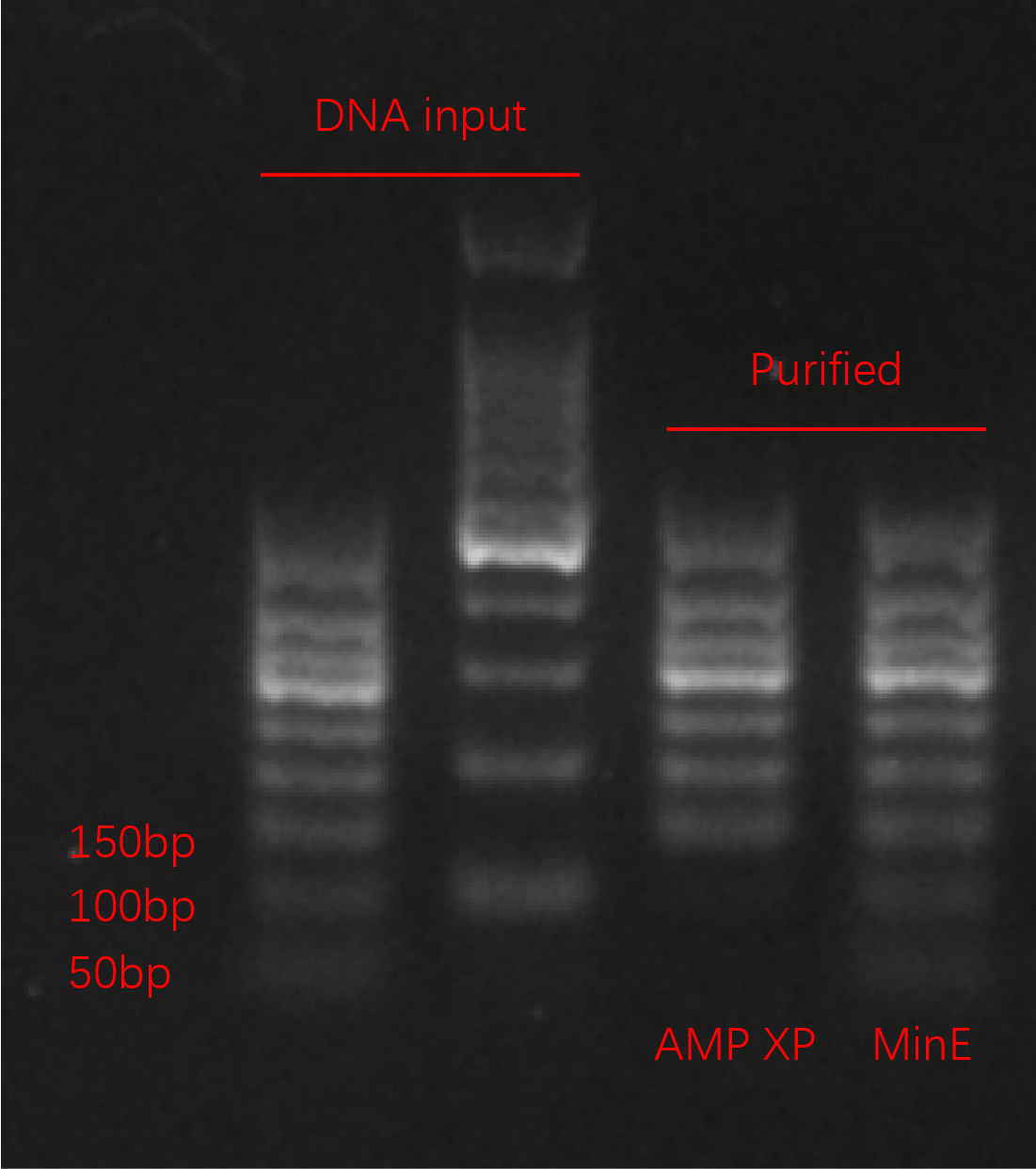


**Fig. S2** DNA library prepared using AMPure XP (Beckman Coulter, USA, lot 19936700, used in our other study).


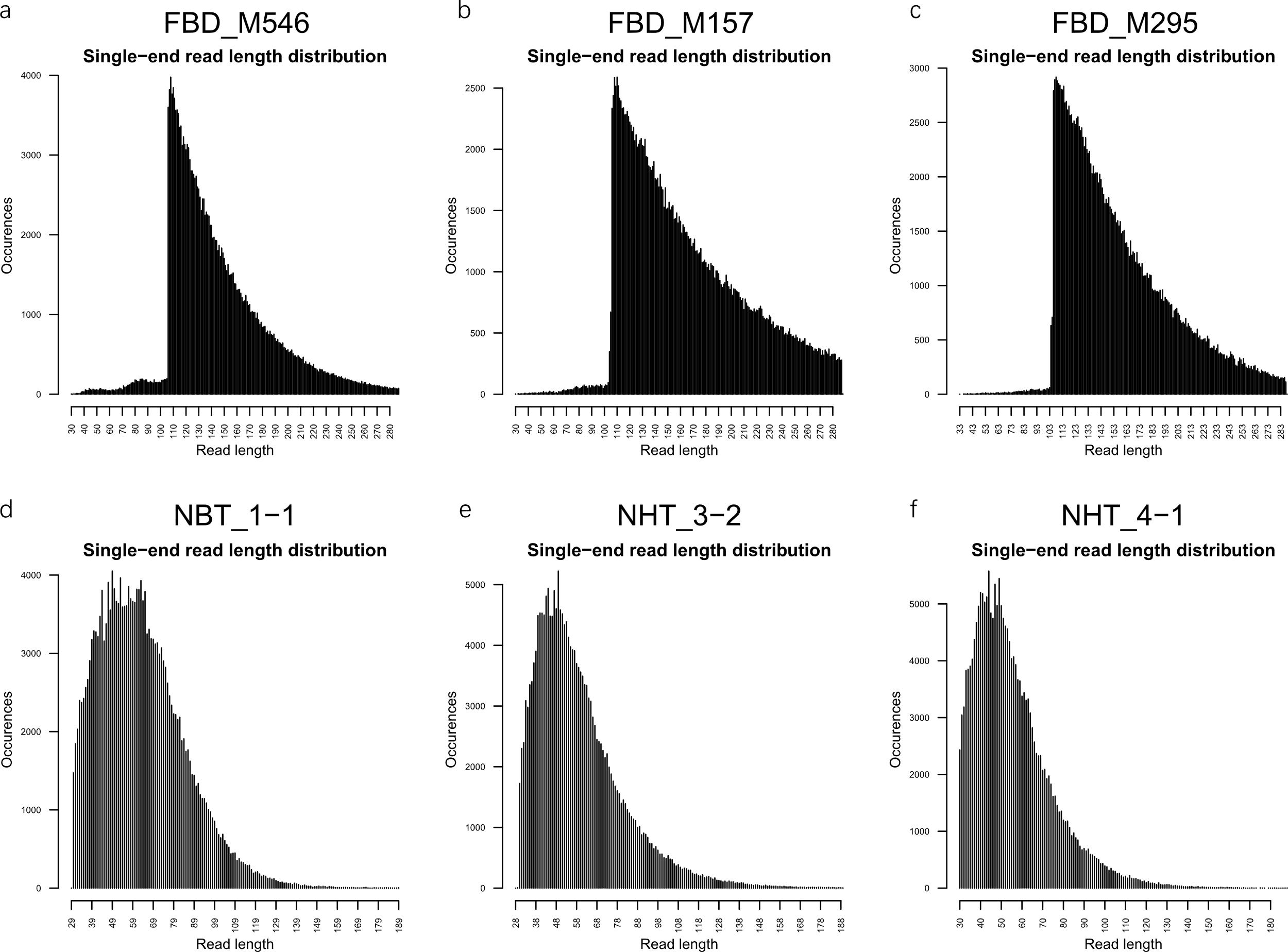


**Fig. S3** Read length frequency analysed by mapDamage. (a-c) Three DNA libraries with top coverage prepared in this study using AMPure XP(Beckman Coulter, USA, lot 19820000). (d-f) Three DNA libraries prepared in our other study with remain reagents unchanged.


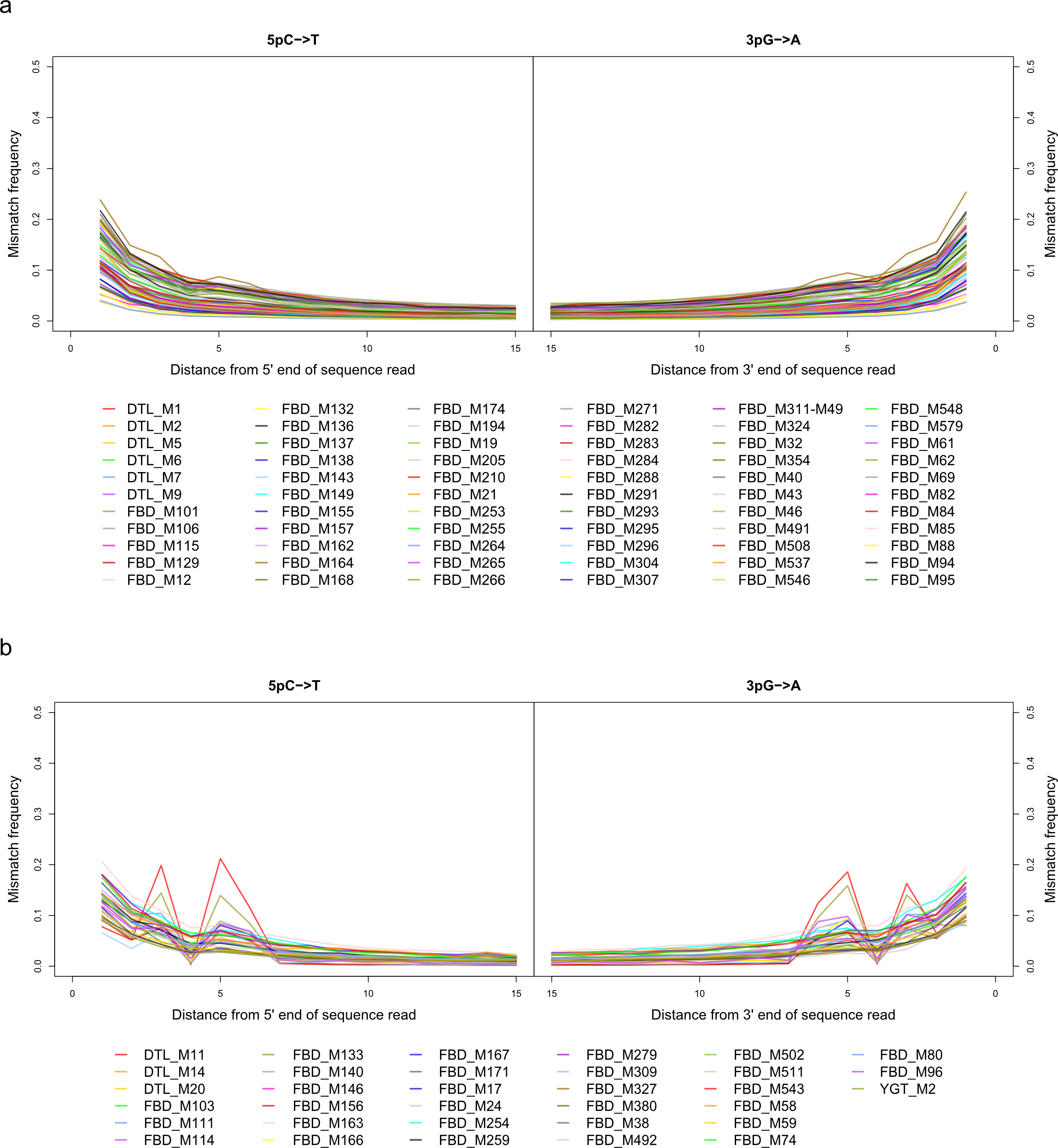


**Fig. S4 Ancient DNA damage patterns.** Nucleotide misincorporation patterns caused by cytosine (C)-to-thymine (T) deamination in ancient DNA sequences. (a) Ancient DNA damage patterns of high coverage samples. (b) Ancient DNA damage patterns of low coverage samples.


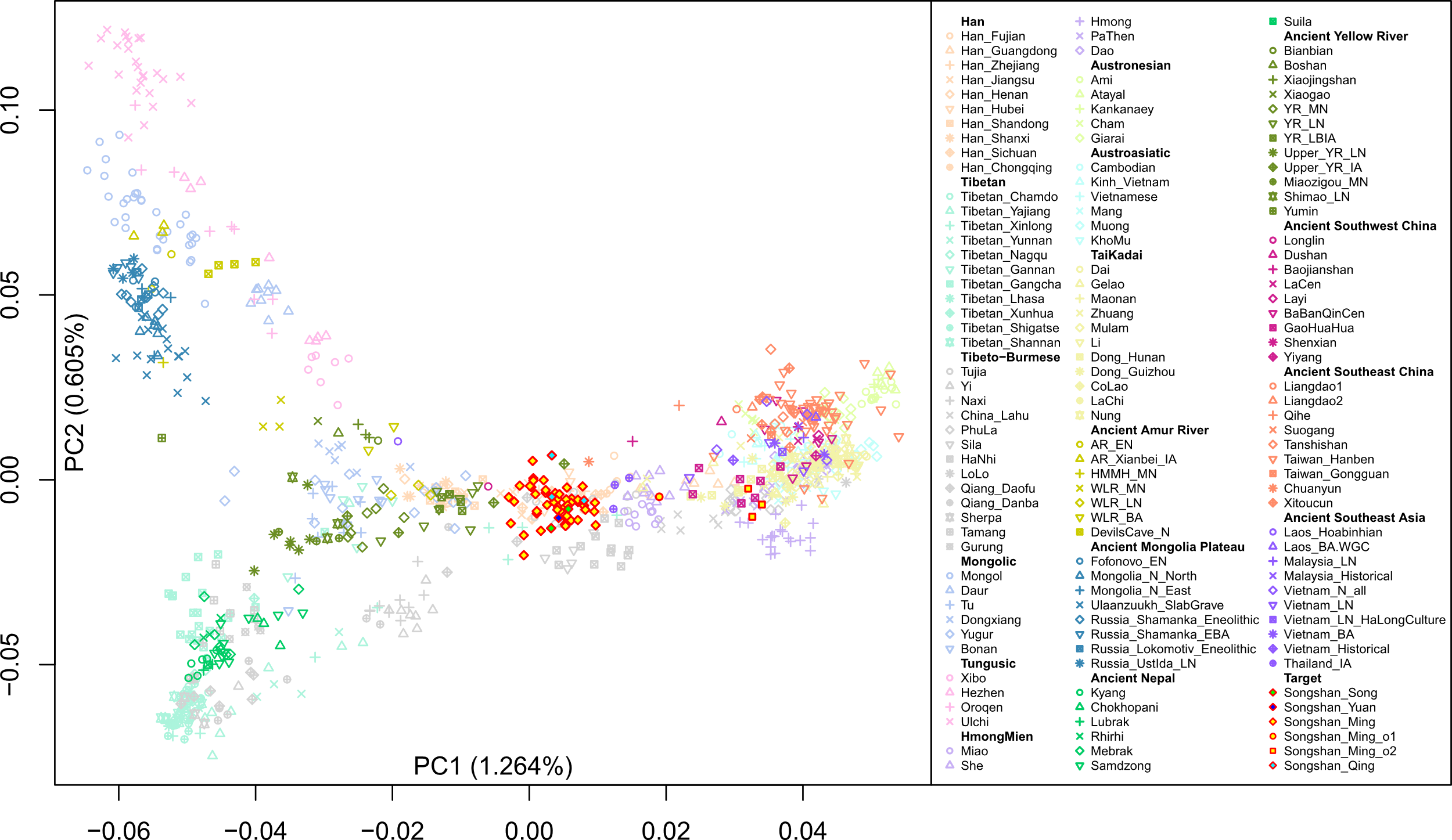


**Fig. S5 Principal Component Analysis (PCA)**. PCA result of Songshan individuals cluster by different time period with present-day and ancient East Asians.


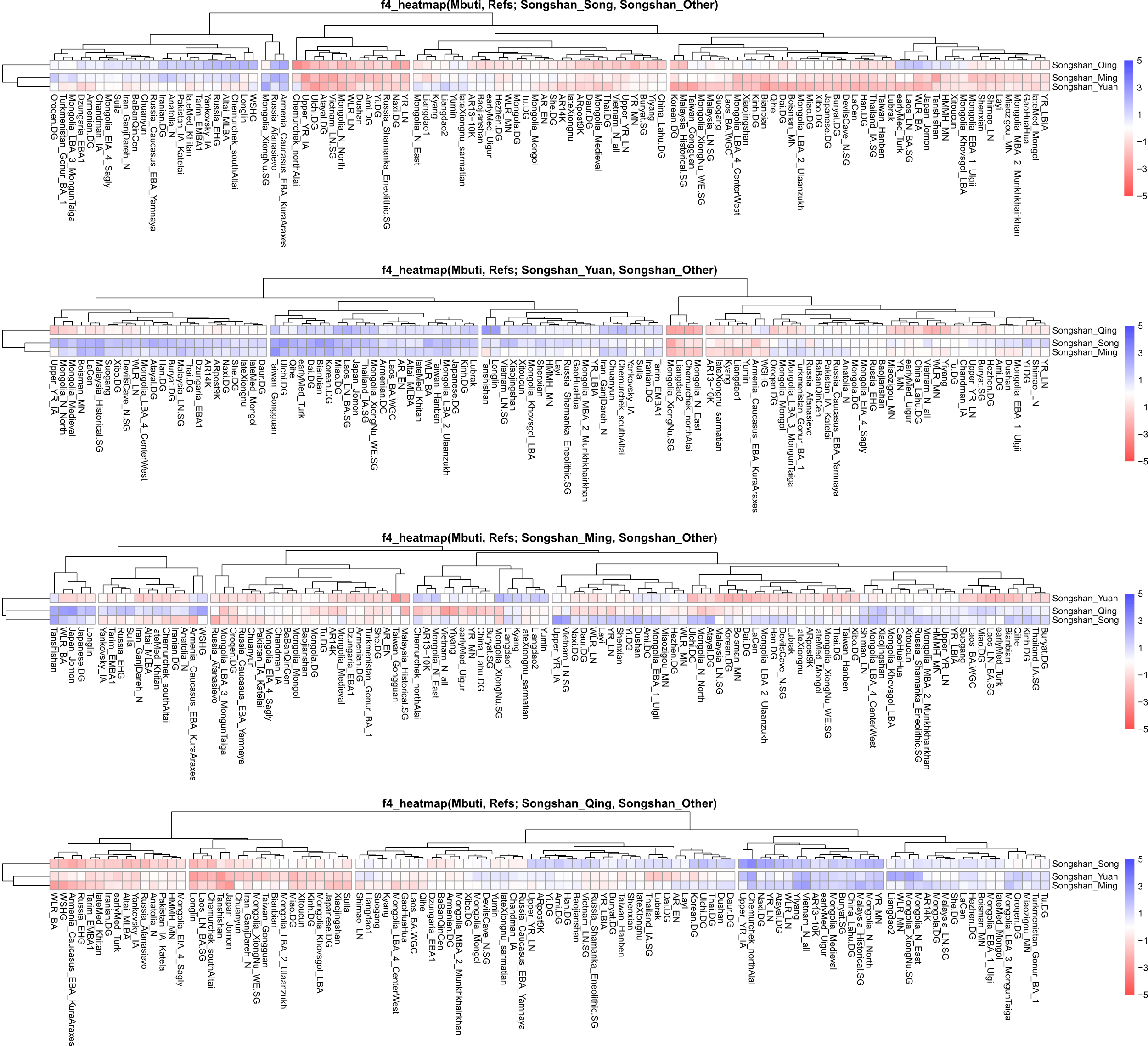


**Fig. S6 Heatmap result of *f_4_*-statistics.** *F*-statistics in the form of *f_4_*(Mbuti, References; Songshan1, Songshan2) shows the genetic consistency of Songshan populations from different time period.


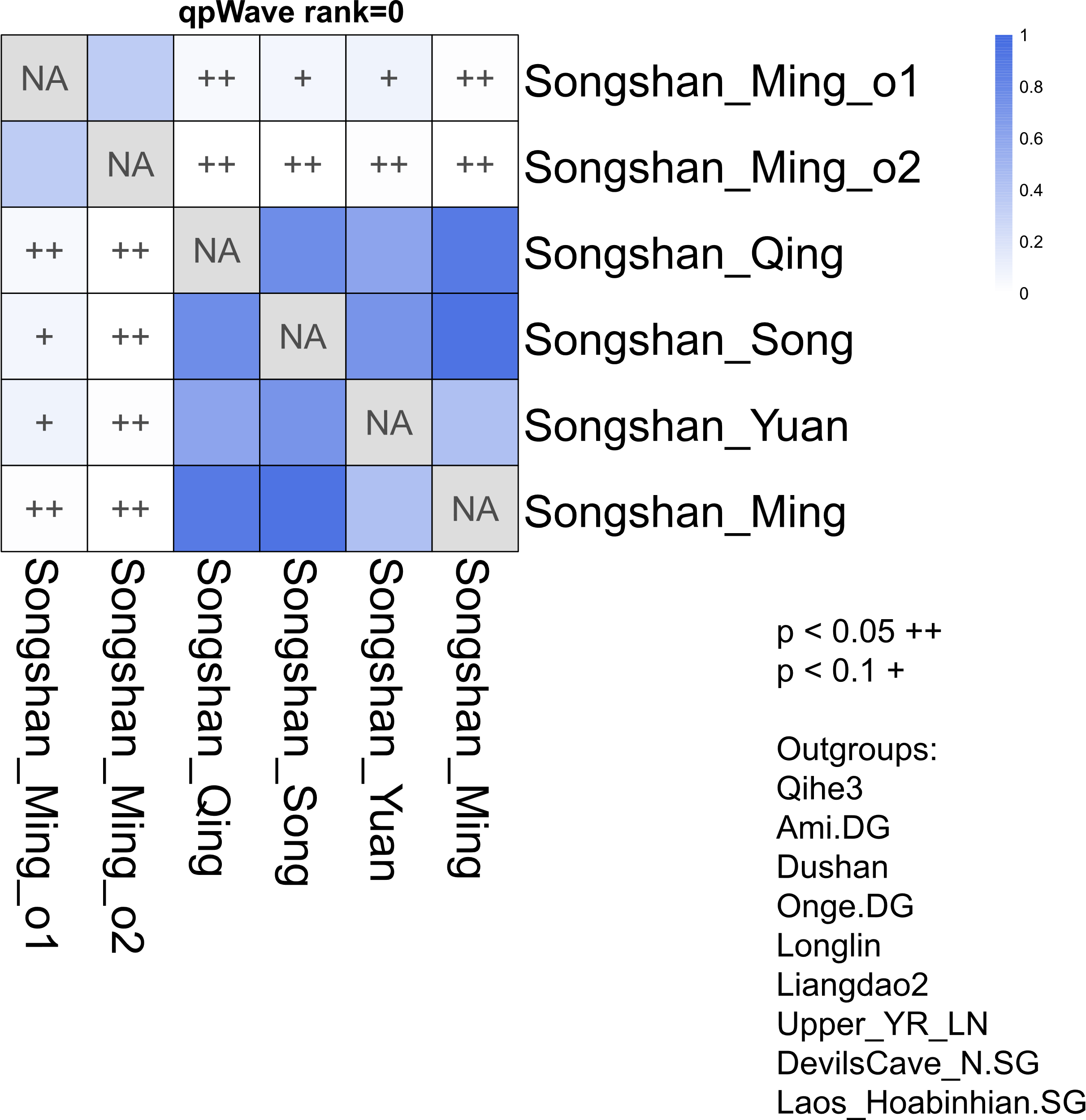


**Fig. S7 Result of pairwise qpWave analysis.** Pairwise qpWave analysis of Songshan populations cluster by different time period.


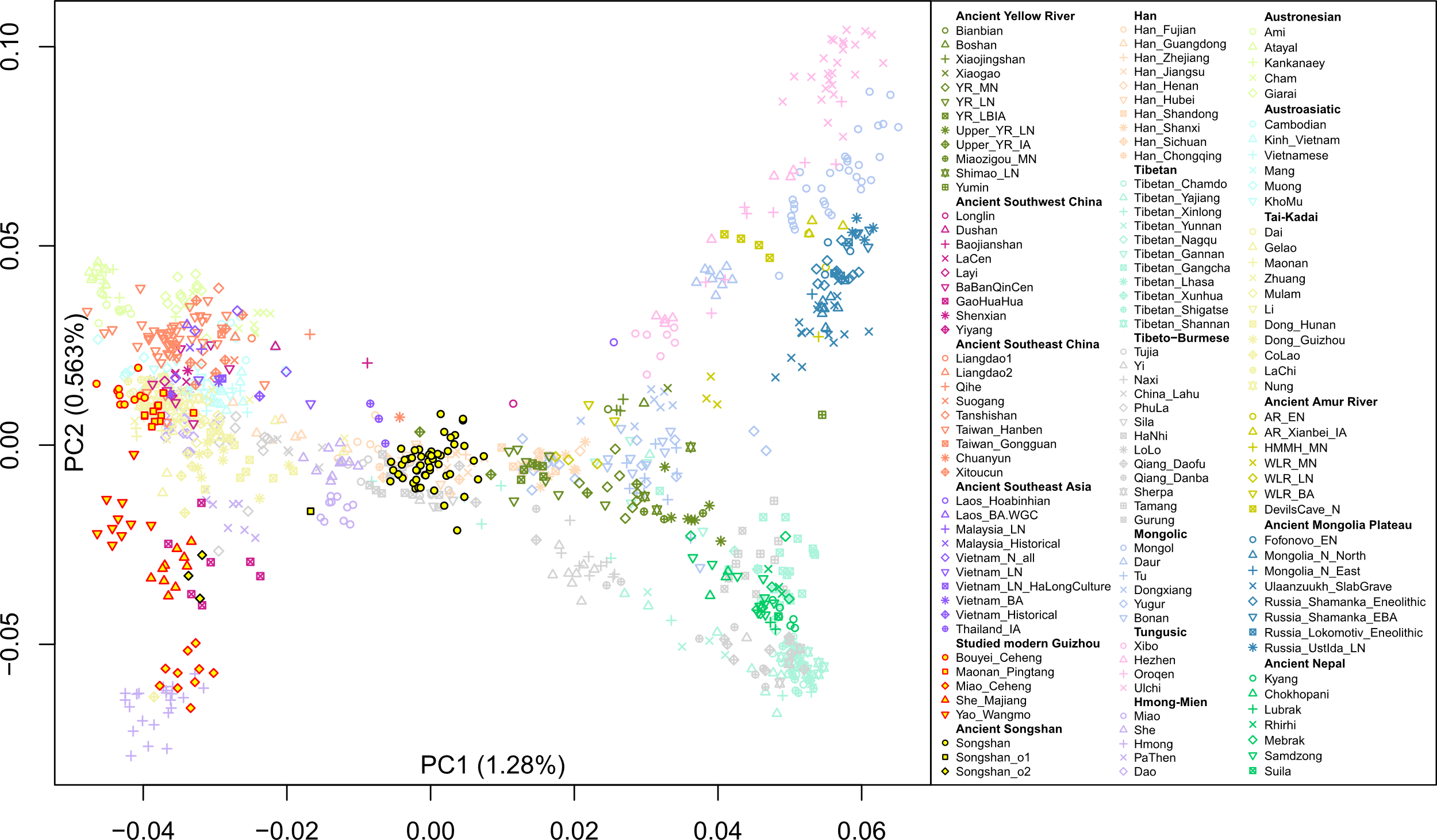


**Fig. S8 Principal Component Analysis (PCA)**. PCA result of Songshan populations and studied modern Guizhou populations with present-day and ancient East Asians.


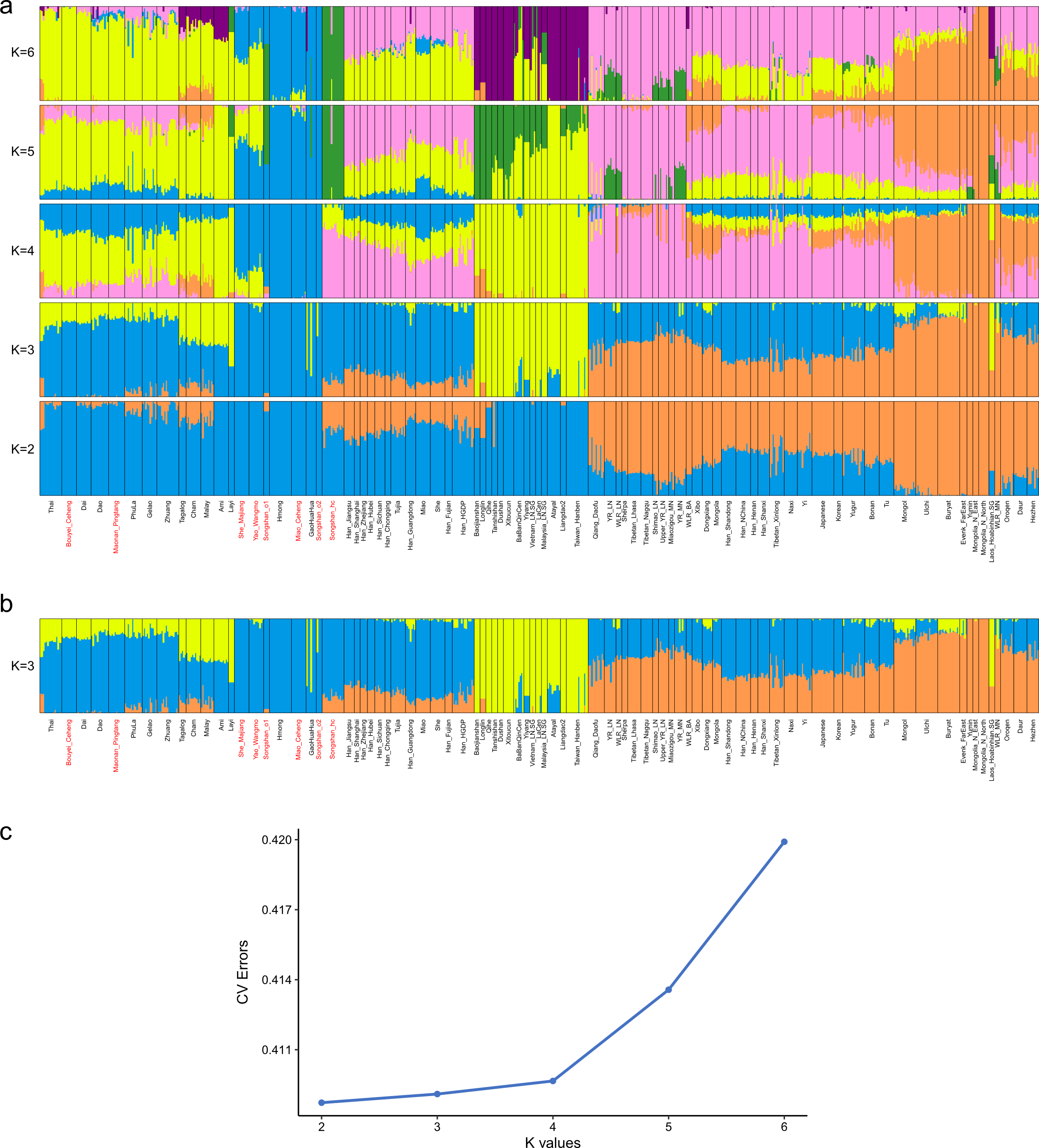


**Fig. S9 Admixture ancestry estimation based on model-based unsupervised ADMXITURE analysis.** (a) The full result, (b) K=3 and (c) cross-validation errors of model-based unsupervised ADMIXTURE analysis. We ran five-fold cross-validation with 100 replicates bootstrapping.


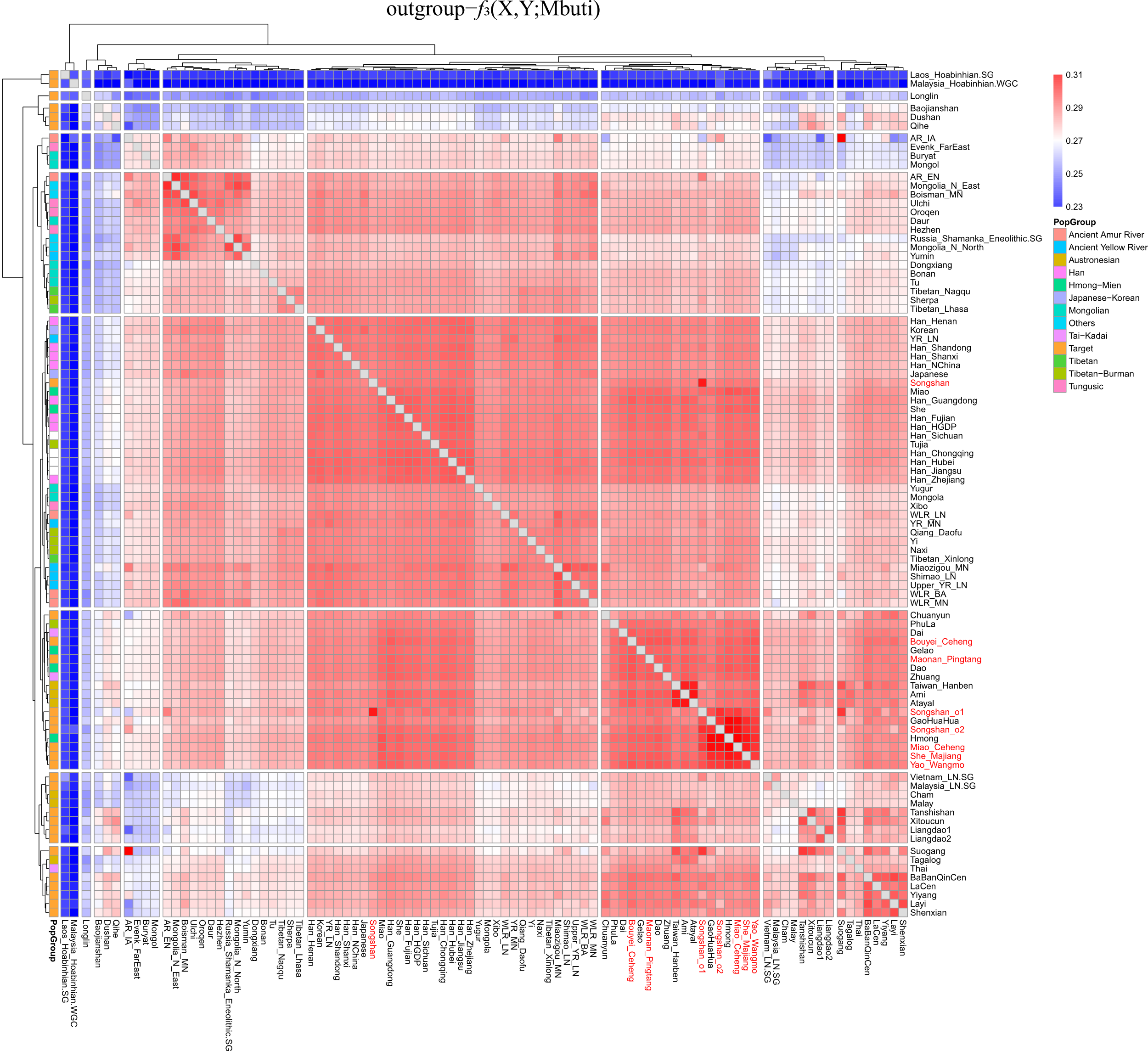


**Fig. S10 Heatmap of pairwise outgroup-*f_3_* test.** Pairwise outgroup-*f_3_* was performed to test the shared genetic drift between Songshan and modern Guizhou populations with ancient and modern East Asians.


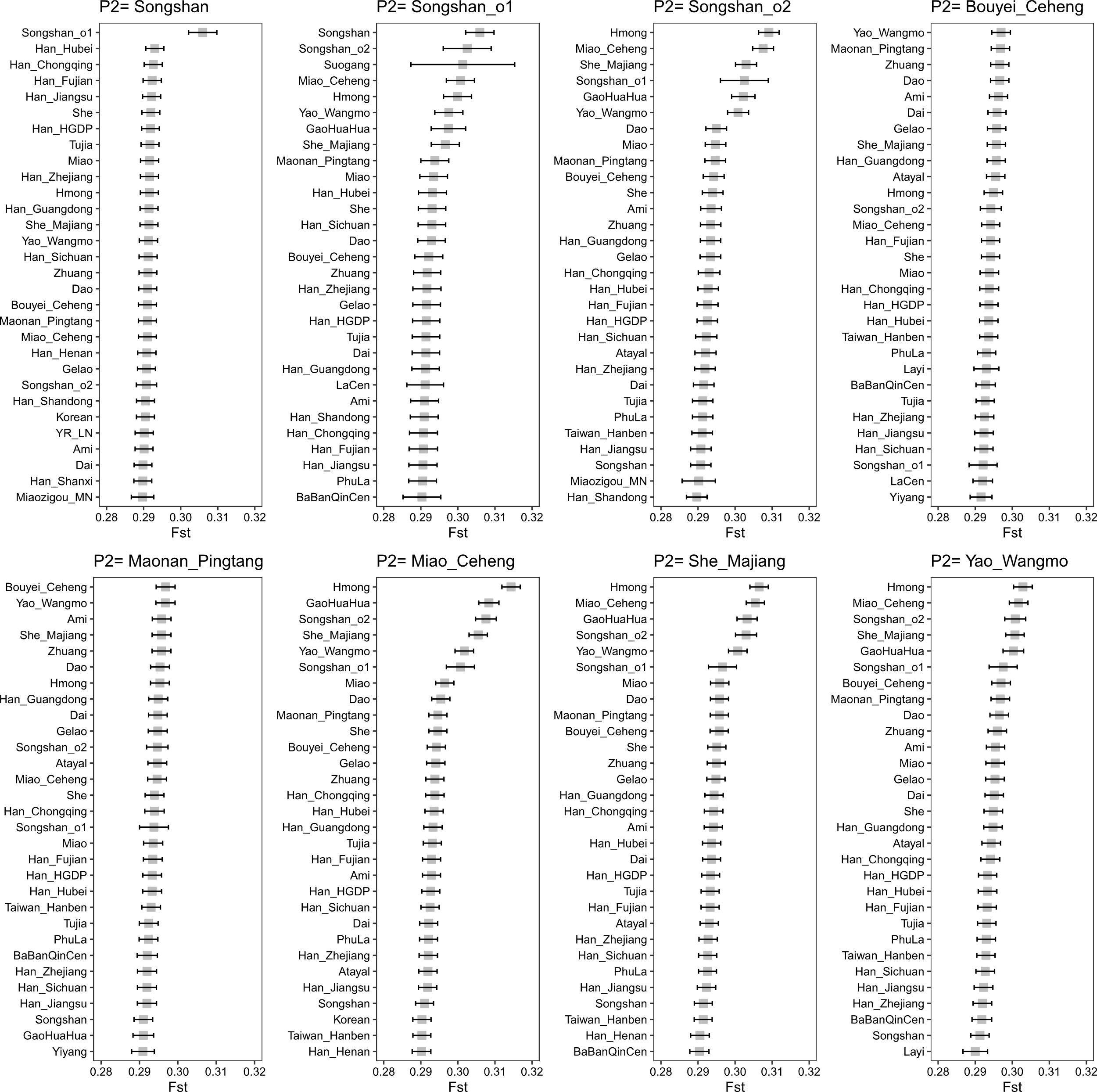


**Fig. S11 Barplot results of outgroup-*f_3_* test.** Outgroup-*f_3_* tests in the format of *f*_3_(X, Y; Mbuti) show the genetic affinity of Songshan and studied modern Guizhou populations with Hmong-Mien and Tai-Kadai populations.

# References

1. Ramsey CB, Lee S. Recent and Planned Developments of the Program Oxcal. *Radiocarbon*. 2013; **55**: 720-730.

2. Reimer PJ, Austin WEN, Bard E *et al.* The Intcal20 Northern Hemisphere Radiocarbon Age Calibration Curve (0-55 Cal Kbp). *Radiocarbon*. 2020; **62**: 725-757.

3. Knapp M, Clarke AC, Horsburgh KA *et al.* Setting the stage - Building and working in an ancient DNA laboratory. *Ann Anat*. 2012; **194**(1): 3-6. doi: 10.1016/j.aanat.2011.03.008

4. Shapiro B, Hofreiter M. Ancient DNA. *Princeton Guide to Evolution*. 2012: 475-481.

5. Meyer M, Kircher M. Illumina sequencing library preparation for highly multiplexed target capture and sequencing. *Cold Spring Harb Protoc*. 2010; **2010**(6): pdb prot5448. doi: 10.1101/pdb.prot5448

6. Rohland N, Mallick S, Mah M *et al.* Three assays for in-solution enrichment of ancient human DNA at more than a million SNPs. *Genome Res*. 2022; **32**(11-12): 2068-2078. doi: 10.1101/gr.276728.122

7. Schubert M, Lindgreen S, Orlando L. AdapterRemoval v2: rapid adapter trimming, identification, and read merging. *BMC Res Notes*. 2016; **9**: 88. doi: 10.1186/s13104-016-1900-2

8. Li H, Durbin R. Fast and accurate short read alignment with Burrows-Wheeler transform. *Bioinformatics*. 2009; **25**(14): 1754-1760. doi: 10.1093/bioinformatics/btp324

9. Peltzer A, Jager G, Herbig A *et al.* EAGER: efficient ancient genome reconstruction. *Genome Biol*. 2016; **17**: 60. doi: 10.1186/s13059-016-0918-z

10. Poplin R, Ruano-Rubio V, DePristo MA *et al.* Scaling accurate genetic variant discovery to tens of thousands of samples. *bioRxiv*. 2018: 201178. doi: 10.1101/201178

11. Skoglund P, Northoff BH, Shunkov MV *et al.* Separating endogenous ancient DNA from modern day contamination in a Siberian Neandertal. *Proc Natl Acad Sci U S A*. 2014; **111**(6): 2229-2234. doi: 10.1073/pnas.1318934111

12. Renaud G, Slon V, Duggan AT *et al.* Schmutzi: estimation of contamination and endogenous mitochondrial consensus calling for ancient DNA. *Genome Biol*. 2015; **16**: 224. doi: 10.1186/s13059-015-0776-0

13. Korneliussen TS, Albrechtsen A, Nielsen R. ANGSD: Analysis of Next Generation Sequencing Data. *Bmc Bioinformatics*. 2014; **15**: 356. doi: 10.1186/s12859-014-0356-4

14. Weissensteiner H, Pacher D, Kloss-Brandstatter A *et al.* HaploGrep 2: mitochondrial haplogroup classification in the era of high-throughput sequencing. *Nucleic Acids Res*. 2016; **44**(W1): W58-W63. doi: 10.1093/nar/gkw233

15. Ralf A, Montiel Gonzalez D, Zhong K *et al.* Yleaf: Software for Human Y-Chromosomal Haplogroup Inference from Next-Generation Sequencing Data. *Mol Biol Evol*. 2018; **35**(5): 1291-1294. doi: 10.1093/molbev/msy032

16. Lipatov M, Sanjeev K, Patro R *et al.* Maximum Likelihood Estimation of Biological Relatedness from Low Coverage Sequencing Data. *bioRxiv*. 2015: 023374. doi: 10.1101/023374

17. Manichaikul A, Mychaleckyj JC, Rich SS *et al.* Robust relationship inference in genome-wide association studies. *Bioinformatics*. 2010; **26**(22): 2867-2873. doi: 10.1093/bioinformatics/btq559

18. Mallick S, Micco A, Mah M *et al.* The Allen Ancient DNA Resource (AADR) a curated compendium of ancient human genomes. *Sci Data*. 2024; **11**(1): 182. doi: 10.1038/s41597-024-03031-7

19. Patterson N, Price AL, Reich D. Population structure and eigenanalysis. *Plos Genetics*. 2006; **2**(12): e190. doi: 10.1371/journal.pgen.0020190

20. Chang CC, Chow CC, Tellier LC *et al.* Second-generation PLINK: rising to the challenge of larger and richer datasets. *Gigascience*. 2015; **4**: 7. doi: 10.1186/s13742-015-0047-8

21. Peter BM. Admixture, Population Structure, and F-Statistics. *Genetics*. 2016; **202**(4): 1485-1501. doi: 10.1534/genetics.115.183913

22. Lawson DJ, Lucy VD, Daniel F. A tutorial on how not to over-interpret STRUCTURE and ADMIXTURE bar plots. *Nat Commun*. 2018; **9**(1): 3258. doi: 10.1038/s41467-018-05257-7

23. Alexander DH, Novembre J, Lange K. Fast model-based estimation of ancestry in unrelated individuals. *Genome Res*. 2009; **19**(9): 1655-1664. doi: 10.1101/gr.094052.109

24. Patterson N, Moorjani P, Luo Y *et al.* Ancient admixture in human history. *Genetics*. 2012; **192**(3): 1065-1093. doi: 10.1534/genetics.112.145037

25. Harney E, Patterson N, Reich D *et al.* Assessing the performance of qpAdm: a statistical tool for studying population admixture. *Genetics*. 2021; **217**(4). doi: 10.1093/genetics/iyaa045

26. Jizhong S. A Study on Han immigrants in Guizhou. *Guizhou Culture And History*. 1990; **01**: 26-33.

27. Fu Q, Hajdinjak M, Moldovan OT *et al.* An early modern human from Romania with a recent Neanderthal ancestor. *Nature*. 2015; **524**(7564): 216-+. doi: 10.1038/nature14558

28. Haak W, Lazaridis I, Patterson N *et al.* Massive migration from the steppe was a source for Indo-European languages in Europe. *Nature*. 2015; **522**(7555): 207-+. doi: 10.1038/nature14317

29. Mathieson I, Lazaridis I, Rohland N *et al.* Genome-wide patterns of selection in 230 ancient Eurasians. *Nature*. 2015; **528**(7583): 499-503. doi: 10.1038/nature16152

30. Mallick S, Micco A, Mah M *et al.* The Allen Ancient DNA Resource (AADR): A curated compendium of ancient human genomes. *bioRxiv*. 2023. doi: 10.1101/2023.04.06.535797
